# Incidence, Characteristics, and Risk Factors of Post‐Radiosurgery Headaches: A Prospective Observational Study

**DOI:** 10.1111/cns.70344

**Published:** 2025-03-18

**Authors:** Shaobo Xiao, Jiayi Liu, Yunmo Liu, Guangshuang Lu, Yan Chang, Jinjing Zhao, Wenjie Su, Xinghao Guo, Nan Gao, Xiufen Zhang, Ke Liu, Zhen Zhang, Shengyuan Yu, Longsheng Pan, Ruozhuo Liu

**Affiliations:** ^1^ Medical School of Chinese PLA Beijing China; ^2^ Department of Neurology, International Headache Center The First Medical Center of Chinese PLA General Hospital Beijing China; ^3^ 920th Hospital of Joint Logistics Support Force of People's Liberation Army of China Kunming Yunnan China; ^4^ Department of Neurosurgery The First Medical Center of PLA General Hospital Beijing China; ^5^ Department of Pediatrics The Lu'an Hospital Affiliated to Anhui Medical University, the Lu'an People's Hospital Lu'an China; ^6^ Department of Neurology The 305 Hospital of the People's Liberation Army Beijing China; ^7^ School of Medicine Nankai University Tianjin China

**Keywords:** Cyberknife, diagnostic criteria, headache, radiosurgery, risk factors

## Abstract

**Aims:**

This prospective observational study aimed to characterize the incidence, clinical features, and risk factors of headaches following CyberKnife radiosurgery (CKRS) in patients with intracranial pathologies.

**Methods:**

In a prospective observational study conducted from January 2022 to January 2023, we enrolled consecutive patients who underwent CKRS. Patients completed headache‐related questionnaires developed based on the International Classification of Headache Disorders (ICHD‐3) guidelines at 24 h, 1 week, and 3 months post‐radiosurgery. The incidence of CKRS‐related headaches was determined, and the link between risk factors and outcomes was analyzed.

**Results:**

Of 153 patients (female 58.2%; mean age 47.7 ± 14.8 years), all completed a 3‐month follow‐up. Among 153 patients, 61 (39.9%) developed post‐CKRS headaches, with 83.6% reporting peak intensity within 2 weeks post‐procedure. Fifty (32.7%) developed headaches within 2 weeks, resolving within 3 months. A strong temporal association between headache onset and CKRS supports a causal relationship. Multivariate Cox regression analysis identified female sex (HR = 2.16, 95% CI = 1.14–4.11, *p* = 0.019), younger age (HR = 0.97 per year, *p* = 0.006), absence of prior craniocerebral surgery (HR = 0.55, *p* = 0.046), and multiple lesions (HR = 2.28, *p* = 0.047) as independent risk factors. Although headaches were more frequently observed following radiation targeting the basal ganglia and thalamus, this association lacked statistical significance (*p* > 0.05).

**Conclusions:**

Headaches attributed to brain radiosurgery constitute a significant yet overlooked clinical issue, warranting increased focus from surgical teams to deliver improved and tailored treatment.

## Graphical abstract

Based on prospective observations of the characteristics of post‐radiosurgery headache, supplementary diagnostic criteria have been proposed. Female gender, younger age, no prior craniocerebral surgery, and multiple lesions are identified as risk factors for the occurrence of post‐radiosurgery headache.

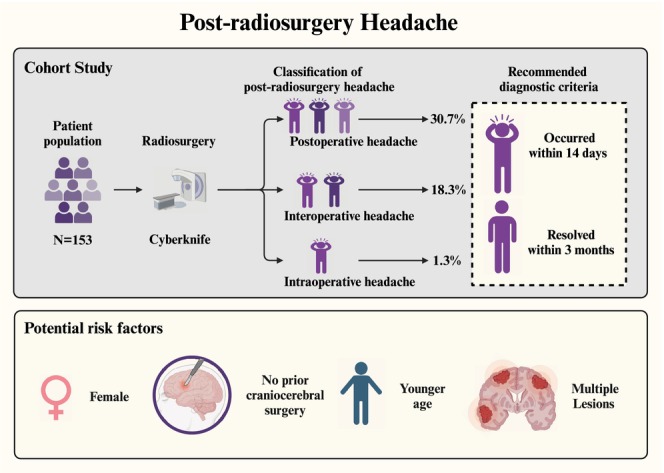

## 1 Introduction

Radiosurgery stands as an effective therapeutic approach for various brain pathologies, including arteriovenous malformations (AVMs), meningiomas, brain tumors, and metastases [1]. It is minimally invasive but can lead to complications [2, 3, 4], including post‐radiosurgery headaches [5, 6], which are not well documented.

A notable instance dates back to 1997, where a migraine‐like headache emerged 15 months post gamma knife radiosurgery in AVM patients [7]. However, this case remains singular, and the extended 15‐month interval complicates establishing a direct link to radiosurgery. Furthermore, accounts of cluster headache‐like symptoms emerging 3 months post‐radiosurgery have been documented [8]. Typically, these individual case reports highlight severe headaches that significantly impede daily functioning, potentially overshadowing cases of milder headaches.

According to the International Classification of Headache Disorders (ICHD‐3), headache attributed to radiosurgery of the brain (HARB) is defined as a new‐onset headache developing within 7 days post‐procedure and resolving within 3 months (Table 1) [9]. However, the incidence, severity, and duration of post‐radiosurgery headaches among neurosurgical patients remain unclear due to a lack of well‐designed clinical and epidemiological studies. Reports vary, suggesting a prevalence from 21 out of 30 cases to just one out of 25 patients [6].

**TABLE 1:** Diagnostic criteria of headache attributed to radiosurgery of the brain in ICHD‐3.

Section A5.7: Headache attributed to radiosurgery of the brain Diagnostic criteria:
A. Any new headache fulfilling criterion C
B. Radiosurgery of the brain has been performed
C. Evidence of causation demonstrated by both of the following:
1. headache has developed within 7 days after radiosurgery
2. headache has resolved within 3 months after radiosurgery
D. Not better accounted for by another ICHD‐3 diagnosis.

Abbreviation: ICHD‐3, the third edition of the International Classification of Headache Disorders.

Our study aimed to comprehensively investigate post‐radiosurgery‐related headaches in patients undergoing CyberKnife radiosurgery (CKRS) and to shed light on the factors associated with them.

## 2 Methods

### 2.1 Study Design

This prospective, observational, single‐center study investigated headache complications after stereotactic CKRS at the Functional Neurosurgery Department of the Chinese PLA General Hospital. For each eligible patient, we performed postoperative follow‐ups at multiple intervals after CKRS to mitigate recall bias: immediately and 24 h after each fraction, as well as 7 days, 14 days, and 3 months after the final fraction. Follow‐ups immediately and 24 h after CKRS were conducted face‐to‐face, while subsequent assessments were conducted via phone. Prior to undergoing radiotherapy, we provided patients with detailed instructions and asked them to maintain a headache diary to personally record their headache experiences. This diary included information such as the date of headache occurrence and the characteristics of the headache, such as duration, location, and intensity. The study protocol received approval from the Institutional Review Board of the Chinese PLA General Hospital (Approval No. S2022‐536‐01).

### 2.2 Participants

Eligible participants included adults (≥ 18 years) undergoing CKRS for intracranial lesions. Individuals with a history of headache were included if they experienced no more than three attacks within 1 week and had no headache episodes within 24 h preceding radiosurgery.

Exclusion criteria included the use of headache‐preventive medications 1 month before radiosurgery, a history of craniotomy within 3 months prior to radiotherapy, traumatic brain injury, cerebral hemorrhage, epilepsy, severe psychological disorders, or other neurological conditions significantly associated with the onset of new headaches. Additionally, patients presenting with severe epilepsy, hemiplegia, aphasia, or other severe neurological manifestations post‐radiosurgery were excluded. Follow‐up brain MRI was mandatory for all patients 3 months after CKRS; those displaying hemorrhage, edema, tumor recurrence, or other significant brain changes postradiation or failing to undergo follow‐up MRI were excluded from the study.

To determine the appropriate sample size, a calculation was implemented using the “Cox Regression” module in the PASS15.0 sample size calculation software, yielding the following results: Cox regression of the log hazard ratio on a covariate with a standard deviation of 5.1270, based on a sample of 147 observations, achieved 90% power at a significance level of 0.05000 to detect a regression coefficient of 0.0953. The sample size was adjusted to an anticipated event rate of 0.3000. Considering potential attrition in our cohort, 200 consecutive patients treated by stereotactic radiosurgery using the CK system (Accuray Inc., Sunnyvale, CA) between January 2022 and June 2023 were included. All participants provided written or oral informed consent.

### 2.3 CyberKnife Technique

Prior to treatment, all patients underwent planning computed tomography (CT) (Siemens, Forchheim, Germany) and 3.0 T magnetic resonance imaging (MRI; Siemens, Erlangan, Germany) with a slice thickness of 1 mm. Rigid fusion registration between T1‐weighted contrast‐enhanced MRI sequences and CT scans was performed using MIM Maestro 6.5.4 image processing software (MIM Software Inc., Cleveland, Ohio, USA). The gross tumor volume (GTV) was determined from the fused image, and the planning target volume (PTV) was defined as the region extending 1.5 mm beyond the GTV. Treatment plans were tailored based on tumor size, its proximity to critical structures, and prior radiation history. All patients received stereotactic radiotherapy (SRT) using the CK system (Accuray Inc., Sunnyvale, CA, USA).

Both single‐fraction and multi‐fractionated cranial radiosurgery were employed, with fractionation involving the division of the total dose into multiple daily administrations. The biologically effective dose (BED) was selected as the parameter for fractionated schemes with respect to the reaction dose. The BED was calculated according to the value of α/β (10 Gy, BED10) using the formula BED = D (1 + d/[α/β]), where D represents the total delivered dose, and d denotes the dose per fraction [10].

### 2.4 Clinical Data and Headache Questionnaire

Demographic information (age, sex, educational level, height, weight, current smoking and drinking status, medical history of hypertension and diabetes, headache history, and surgical history) and surgical details (clinical diagnosis, lesion number and location, radiation site, BED, brainstem average dose, brainstem maximum dose, radiation brainstem volume, target average dose, target maximum dose, and PTV) were collected for each patient.

Smoking history: Defined as having smoked regularly (≥ 1 cigarette per day for more than 6 months). Drinking history: Defined as having consumed alcohol regularly (≥ 1 time per week for more than 6 months). Diabetes and hypertension: Both were diagnosed according to international standards in medical institutions. All the above indicators were dichotomized into “yes” or “no.” Education level: Categorized into two groups based on whether the individual had completed high school or not. Body mass index (BMI): Calculated as weight (kg) divided by the square of height (m^2^).

A questionnaire, developed based on the ICHD‐3 guideline [9], included detailed headache‐related inquiries such as onset time, duration, resolution time, headache characteristics (unilateral or bilateral), location (frontal, temporal, occipital, parietal, or full head), intensity, quality (throbbing, dull, pressing, stabbing, and burning), and associated symptoms (nausea, vomiting, photophobia, phonophobia, and aura). The Numeric Pain Rating Scale (NRS), a segmented numeric version of the visual analog scale (VAS), was employed to assess pain intensity, with participants instructed to select a whole number (0–10 integers) reflecting their pain intensity [11].

### 2.5 Statistical Analysis

Statistical analyses were performed using IBM SPSS Statistics software version 27.0. Continuous data conforming to a normal distribution are presented as mean ± standard deviation (SD), while categorical data are expressed as numbers and percentages. Continuous data not following a normal distribution are reported as median and interquartile range. Differences in means between groups were assessed using independent samples *t*‐test or analysis of variance. Connections between categorical variables were examined using Fisher's exact test or Pearson's chi‐squared test. Headache onset time was utilized as an outcome measure, calculated from treatment initiation. Univariate COX regression analysis was performed to identify potential risk factors, followed by multivariate Cox regression analyses on clinically significant variables (*p* < 0.1 on univariate analysis). Statistical significance was set at *p* < 0.05.

## 3 Results

### 3.1 Patients' Enrollment

**FIGURE 1:**
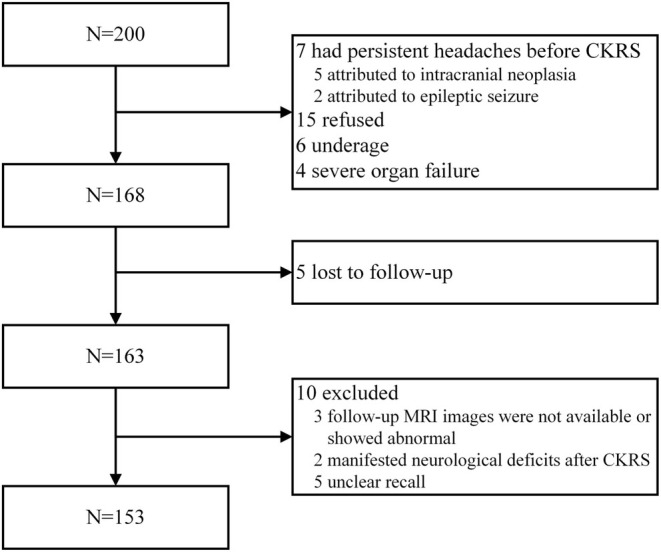
Study enrolment.

A total of 200 consecutive patients underwent CKRS between January 2022 and June 2023, with 153 patients completing the study (Figure 1). Seven patients had persistent headaches before the interventional procedure, attributed to intracranial neoplasia (*n* = 5) or epileptic seizures (*n* = 2). Six underage patients and four individuals with severe organ failure were excluded. Additionally, 15 patients declined participation, and 10 discontinued follow‐ups due to deteriorating health or other reasons. Five patients were uncertain about experiencing headaches during the follow‐up period.

### 3.2 Demographic Information of the Patients

**FIGURE 2:**
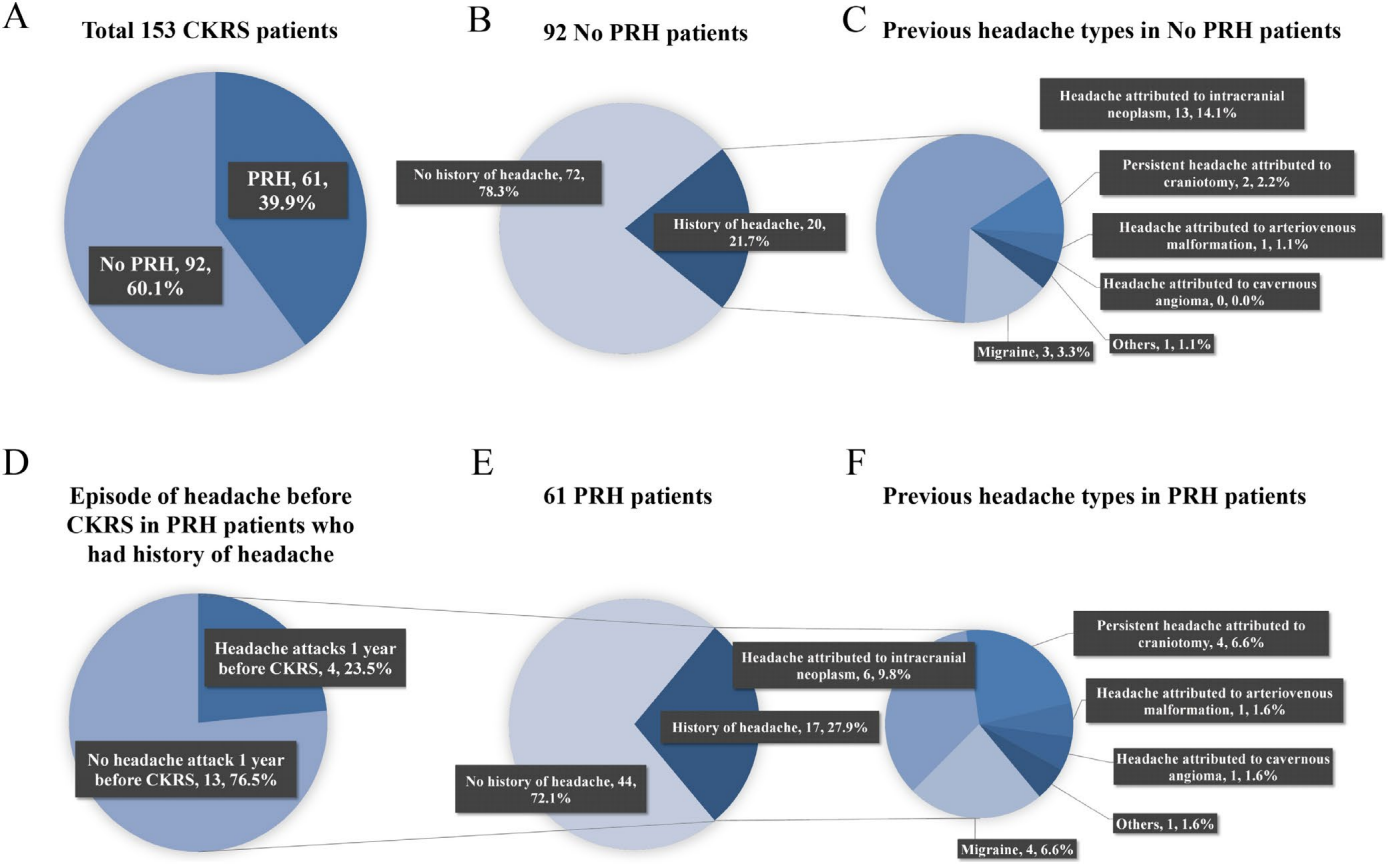
Previous headache conditions among 61 patients with PRH and 92 patients without PRH. (A) 39.9% of patients reported PRH during the 3‐month follow‐up period, and 60.1% did not. (B, C) Among patients who did not experience PRH, 21.7% had a history of previous headaches, and previous headache types are shown. (D–F) Among patients who experienced PRH, 27.9% had a history of previous headaches. Whether they still had headache attacks before CKRS and previous headache types are shown. PRH, post‐radiosurgery headache.

Of the 153 patients (female 58.2%; mean age 47.7 ± 14.8 years), the cumulative headache incidence was 39.9% over 3 months (Figure 2A). Table S1 displays demographic characteristics. A significant difference was noted in age (44.5 ± 13.5 years in HA vs. 49.7 ± 15.4 years in no HA, *p* = 0.034) and smoking history (18.0% smoking in HA vs. 32.6% smoking in no HA, *p* = 0.046) between the headache in/after radiosurgery (HA) and no headache in/after radiosurgery (no HA) groups. However, no significant differences were observed in sex, educational level, BMI, drinking history, history of hypertension and diabetes, or history of brain surgery (Table S1 in Supplement).

### 3.3 Association Between Previous Headache and Headache in/After Radiosurgery

Thirty‐seven patients (24.2%) had a history of headache before CKRS, including 19 headaches attributed to intracranial neoplasm (INH), seven migraines, six persistent headaches attributed to craniotomy (CPH), two headaches attributed to arteriovenous malformation (AVMH), one headache attributed to cavernous angioma (CAH), and two other types of headaches (Figure 2B–F). Among them, 20 (54.1%) did not experience headaches 3 months after CKRS, while 17 (45.9%) did.

Although 37 patients had a history of headache, only seven still had headache attacks before surgery, but their frequency was less than three times a week, and they did not experience headaches 24 h before radiosurgery. Among these, four developed new headaches post‐surgery, which emerged within 7 days of the operation and significantly subsided within 3 months.

Furthermore, patients with a history of headache before surgery demonstrated a higher incidence of headache during or after radiosurgery compared to those without a history, though it was not statistically significant (*p* < 0.05).

### 3.4 Incidence and Characteristics of the Headache

We delineated three distinct headache periods: intraoperative headache (during CKRS), interoperative (between sessions or within 24 h post‐session), and postoperative (1–90 days after final CKRS fraction) (Table S2). Detailed headache characteristics for each patient are outlined in Table S3. The incidence rates were as follows: intraoperative 1.3% (2/153), interoperative 18.3% (28/153), and postoperative 30.7% (47/153), with one patient experiencing both interoperative and postoperative headaches.

During CKRS, 1.3% (2/153) experienced severe, bilateral headaches, with an average onset time of 25.0 ± 7.1 min postradiation, lasting 20.0 ± 14.1 min. Interoperative headaches (18.3%, 28/153) occurred approximately 4.6 ± 4.7 h after CKRS, lasting 8.4 ± 9.6 h/episode, resolving within 11.5 ± 16.9 days post‐CKRS. Most were unilateral (78.6%) and dull (64.3%), with some experiencing nausea (35.7%) and vomiting (28.6%).

Post CKRS, 30.7% (47/153) developed headaches. Among them, 30 patients experienced headaches within 1–7 days post‐treatment, seven patients experienced headaches 8–14 days post‐treatment, and 10 patients experienced headaches 15–90 days post‐treatment. The average durations of each headache episode were 12.4 ± 3.2 h, 8.0 ± 4.2 h, and 27.4 ± 16.3 h, respectively, while the average durations of headache relief were 29.9 ± 12.0 days, 32.0 ± 7.2 days, and 52.6 ± 16.2 days, respectively. Predominantly unilateral and moderate, postoperative headaches had lower nausea/vomiting compared to interoperative headaches.

### 3.5 Diagnostic Criteria of HARB

**FIGURE 3:**
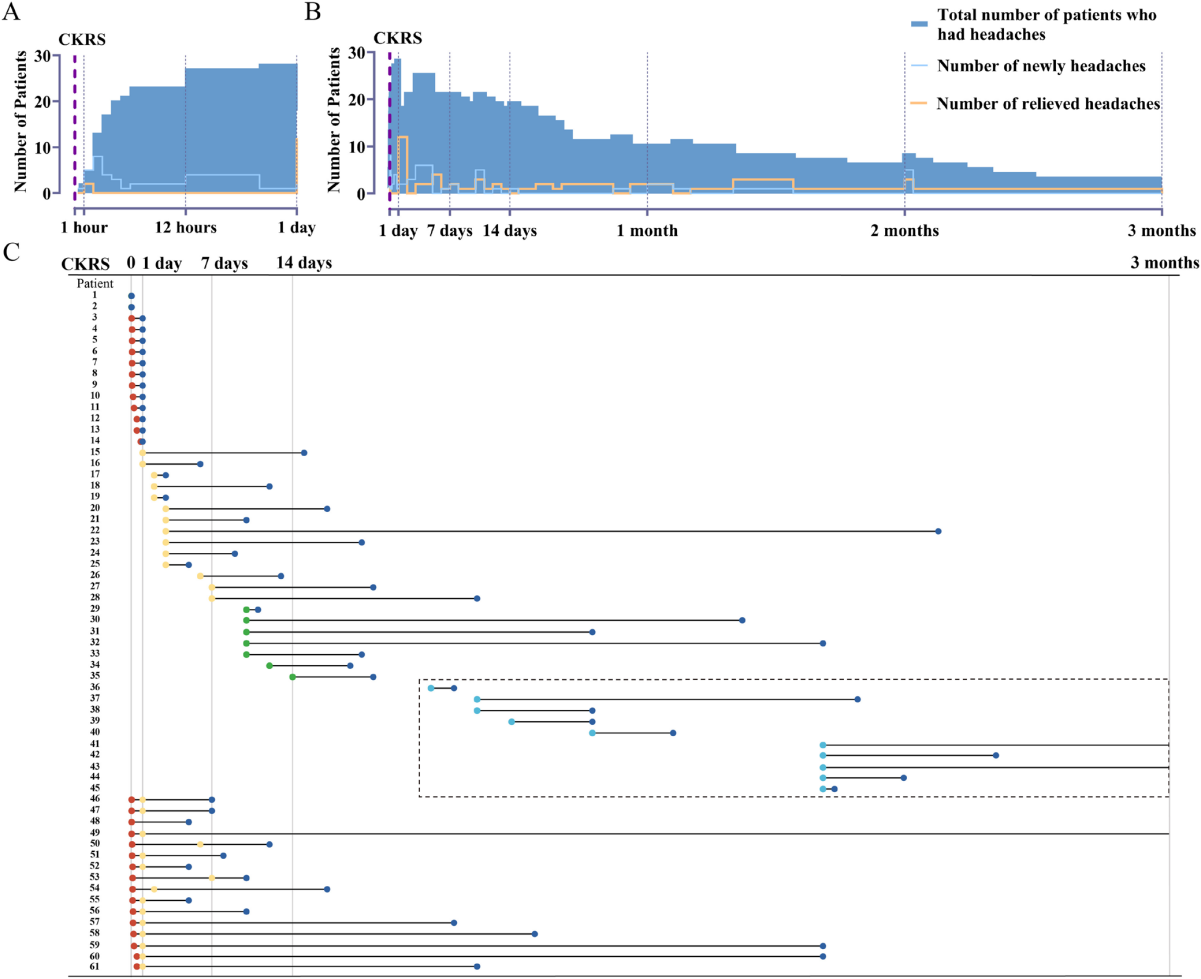
Changes in the number of headache cases over time after CKRS. (A) Changes in the occurrence and resolution of intraoperative and interoperative headaches: The timeline originates from the start of CKRS. (B) Changes in the occurrence and resolution of postoperative headaches: The timeline originates from the end of the last CKRS fraction. (C) The timelines of PTH occurrence and resolution in a total 61 patients. Patients 1 and 2 had intraoperative headaches. Red points represent the start of interoperative headaches; yellow, green, and light blue points represent the start of postoperative headaches; and dark blue points represent the end of headaches. The dashed box indicates patients who experienced headaches for more than 2 weeks after CKRS.

Figure 3 shows the headache onset and relief times for the 61 patients who reported headaches, including 16 patients who experienced both intraoperative and postoperative headaches (Figure 3A). Headache onset was predominately (83.6%) within surgery to 2 weeks after the last fraction, with 95.1% resolving within 90 days (Figure 3B). Most intraoperative headaches resolved post‐surgery, with a notable portion (12/28, 42.9%) experiencing relief on the same day. However, it is worth mentioning that a majority (16/28, 57.1%) also experienced postoperative headaches, all within 7 days following surgery. Few (4.9%) still had headaches 3 months post‐surgery (Figure 3A, No. 41, 43, 49).

The current HARB diagnostic criteria in the ICHD‐3 define it as a headache occurring within 7 days post‐radiosurgery, resolving within 90 days. In our study, 43 patients met these criteria, with an estimated 28.1% incidence. For patients with headaches 7–14 days post‐surgery, we suspect a causal link with CKRS due to the short interval between onset and treatment, and complete relief over time, akin to with those occurring within 1–7 days post‐surgery, supporting their CKRS association.

Conversely, 20% (2/10) of patients with headaches 15–90 days after radiosurgery had no relief within 90 days. These later‐onset headaches differed in severity and symptoms from those appearing 1–7 days post‐surgery, suggesting screening for other possible headache causes, as primary headaches cannot be excluded.

Based on these observations, we propose revising the diagnostic criteria to include headaches during or within 14 days post‐surgery, resolving within 90 days (Table 2). Under these revised criteria, 50 patients developed HARB, an incidence rate of 32.7%.

**TABLE 2:** Recommended diagnostic criteria for headache attributed to radiosurgery of the brain.

Section A5.7: Headache attributed to radiosurgery of the brain Diagnostic criteria:
A. Any new headache fulfilling criterion C‐D
B. Radiosurgery of the brain has been performed
C. Evidence of causation demonstrated by at least one of the following:
1. Headache has developed during radiosurgery of the brain
2. Headache has developed after radiosurgery of the brain within 14 days
D. Headache has resolved within 3 months after radiosurgery
E. Not better accounted for by another ICHD‐3 diagnosis

Abbreviation: ICHD‐3, International Classification of Headache Disorders‐3rd Edition.

Table S4 in Supplement shows demographic and clinical characteristics of the HA and non‐HA groups according to our proposed criteria. Significant differences were found in age and BED between the groups (*p* = 0.041 and *p* = 0.048, respectively; chi‐squared test). No significant differences were found in sex, education level, BMI, smoking/drinking history, hypertension/diabetes, brain surgery history, lesion number, meningeal involvement, brainstem radiation metrics, or target volume between the groups.

### 3.6 Potential Risk Factors Related to Headache

We initially conducted univariate Cox proportional hazards regression analyses to identify predictors of HARB (Table 3). Factors with a *p*‐value < 0.1 (age, history of brain surgery, number of lesions, and BED) were included in multivariate Cox proportional hazards regression analyses. Additionally, considering the potential relevance of sex (*p* = 0.109) and planning target volume (*p* = 0.580) based on discussions with neurologists and neurosurgeons, these factors were also included in the analyses. Our findings indicated that female sex, younger age, no history of brain surgery, and multiple lesions were significant risk factors for HARB (Table 4).

**TABLE 3:** Potential risk factors for HARB in univariate Cox regression analysis.

Variables	HR	95% CI	*p*
Gender			
Female	1.632	0.897–2.967	0.109
Age (years)	0.977	0.956–0.999	0.042
Educational level			
≥ High school	0.804	0.437–1.482	0.485
BMI (kg/m^2^)	0.981	0.907–1.062	0.639
Smoking history			
Yes	0.818	0.416–1.611	0.562
Drinking history			
Yes	0.814	0.413–1.604	0.552
History of headache			
Yes	0.665	0.357–1.240	0.199
History of brain surgery			
Yes	0.510	0.286–0.908	0.022
History of hypertension			
Yes	0.807	0.530–2.264	0.807
History of diabetes			
Yes	1.292	0.310–5.378	0.725
Lesion number			
Multiple	1.876	0.949–3.705	0.070
Lesion involvement			
Meninges	1.085	0.594–1.981	0.792
BED (Gy)	1.024	1.000–1.049	0.052
Brainstem average dose (Gy)	0.959	0.835–1.101	0.549
Brainstem maximum dose (Gy)	0.986	0.952–1.021	0.431
RBV (cm^3^)	1.007	0.955–1.062	0.799
Target average dose (Gy)	0.991	0.956–1.028	0.646
Target maximum dose (Gy)	1.009	0.943–1.080	0.787
PTV (cm^3^)	1.010	0.976–1.044	0.580

Abbreviations: BED, biologically effective dose; CI, confidence interval; HARB, headache attributed to radiosurgery of the brain; HR, hazard ratio; PTV, planning target volume; RBV, radiation brainstem volume.

**TABLE 4:** Potential risk factors for HARB in multivariate Cox regression analyses.

Variables	HR	95% CI	*p*
Gender			
Female	2.159	1.135–4.107	0.019*
Age (years)	0.969	0.947–0.991	0.006*
History of brain surgery			
Yes	0.545	0.301–0.989	0.046*
Lesion number			
Multiple	2.278	1.010–5.135	0.047*
BED (Gy)	1.030	0.999–1.061	0.059
PTV (cm^3^)	0.978	0.937–1.020	0.295

Abbreviations: BED, biologically effective dose; CI, confidence interval; HARB, headache attributed to radiosurgery of the brain; HR, hazard ratio; PTV, planning target volume.

* Significant statistical differences.

### 3.7 Headache and Its Related Brain Regions and Disease Types

**FIGURE 4:**
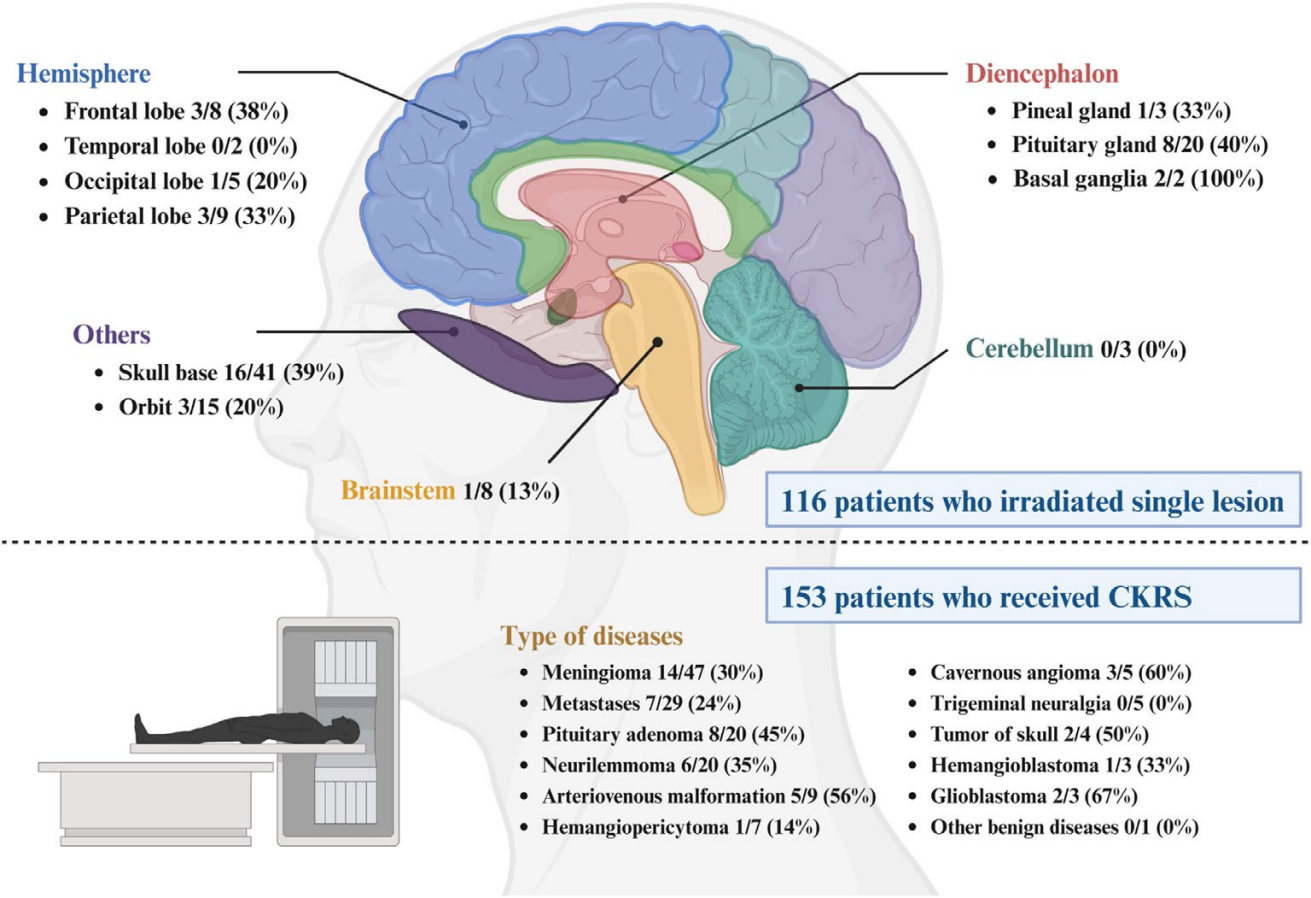
Relationship between the incidence of HARB and location of radiation exposure as well as type of disease.

Our goal was to explore which brain areas are more likely to cause headaches when exposed to radiation, to understand the possible mechanisms underlying headache development. Given the challenge of pinpointing which lesion caused the headache in patients with multiple lesions, we focused on 116 patients (Figure 4) who received treatment for a single lesion.

Our study revealed that two of 20 patients with pituitary tumors reported headaches during radiosurgery, suggesting a possible link between direct pituitary gland stimulation and headache occurrence. Furthermore, six of these 20 patients experienced postoperative headaches, indicating that nearly half of the pituitary tumor patients encountered headaches, implying that the pituitary gland might be a pivotal area for headache manifestation. Notably, both patients who underwent radiotherapy targeting the basal ganglia developed treatment‐associated headaches. Irradiation of the skull base and frontal lobe resulted in headaches in 39% (16/41) and 38% (3/8) of the patients, respectively. None of the three patients who underwent cerebellar radiation and the two patients who received temporal lobe irradiation developed headaches. However, no statistically significant difference was observed between the locations of radiotherapy (cortex, diencephalon, brainstem, cerebellum, or cranial base) and the occurrence of HARB (*p* > 0.05).

Among the 153 patients, glioblastomas had the highest incidence of HARB, followed by cavernous hemangiomas and arteriovenous malformations (Figure 4). However, due to the diversity of diseases and the relatively small number of patients with each disease, no significant statistical difference was found in the correlation between disease type and headache occurrence.

## 4 Discussion

To the best of our knowledge, our study is the first to investigate headaches associated with HARB after CKRS, providing significant clinical insights. We continuously monitored patients post‐CKRS for various conditions, suggesting headaches may relate to radiosurgery itself. We established strict criteria to correlate headaches with radiosurgery, excluding confounding factors, such as patients who frequently experienced headaches before radiosurgery to avoid confusion between pre‐existing headaches and those caused by radiosurgery. Patients who had undergone craniotomy within 3 months prior to radiosurgery were excluded, as headache is a common complication after craniotomy [12], making it difficult to determine the cause of headache in these patients. Additionally, patients with a history of head injury, cerebral hemorrhage, epilepsy, or severe psychiatric symptoms before radiosurgery were excluded, as these conditions might also lead to secondary headaches [13, 14, 15]. Furthermore, through postoperative follow‐up, we excluded all patients who developed serious complications, such as obvious epileptic or other neurological symptoms, which could themselves cause headaches [16]. Patients with imaging evidence of edema [17], hemorrhage [18], or tumor metastasis [19] were also excluded, as their headaches could not be unambiguously attributed to radiosurgery.

Of 61 patients reporting headaches, 50 were diagnosed with headache attributed to brain radiosurgery for the following reasons: (1) they underwent brain radiosurgery, a procedure that may trigger headaches; (2) their headaches emerged within 2 weeks of receiving radiosurgery, indicating a close temporal association; (3) their headaches resolved within 3 months after the final radiosurgery fraction, suggesting that the headaches subsided within a certain period post‐surgery; (4) their headaches exhibited common characteristics; and (5) their headaches were not better accounted for by another diagnosis. Thus, we suggest that headaches attributed to radiotherapy do indeed exist, with an incidence rate of 32.7%. We propose revising the diagnostic criteria for headache attributed to brain radiosurgery in ICHD‐3 patients, as shown in Table 2.

While determining delayed‐onset headaches' cause post‐radiosurgery is challenging, the initial trigger is often presumed to be radiosurgery. We suggest a 2‐week latency may be more appropriate, acknowledging that some headaches may not be related to radiosurgery. For example, Rozen et al. reported a case of migraine‐like symptoms emerging 15 months after gamma knife radiosurgery [7], lacking conclusive evidence linking it to the procedure. Additionally, delayed‐onset secondary headaches can result from brain trauma or craniotomy [20], leading to a classification of headaches into immediate and delayed onset.

Our study found that among seven patients experiencing intermittent headaches within a year before surgery, four developed headaches within 3 months post‐operation (Table S3). Notably, all these patients had a history of episodic migraine. However, the post‐radiosurgery headaches differed significantly from their previous episodes in duration, location, nature, and pain intensity, suggesting a likely linked connection to radiosurgery. Therefore, we propose extending the follow‐up period for these patients to monitor their headache status and assess any changes attributable to radiosurgery.

Individuals with a history of headaches may exhibit a reduced pain threshold [21], potentially explained by central sensitization [22]. As such, patients with a prior history of headaches may be more susceptible to HARB. While our study observed a higher incidence of HARB in patients with preoperative headaches compared to those without a headache history, the study lacked statistical significance, warranting larger‐scale investigations.

Headaches occurring intraoperatively, intraoperatively, or postoperatively exhibit distinct characteristics in nature, severity, accompanying symptoms, and duration, suggesting different underlying mechanisms, such as radiation‐induced stimulation of pain fibers, cerebral edema, and increased intracranial pressure [17, 23], hormonal imbalances, endocrine homeostatic changes, or other radiation‐induced pathological alterations [24, 25].

We observed that pituitary‐related headaches, often severe and accompanied by nausea or vomiting [26], were prevalent among patients undergoing radiosurgery targeting the pituitary gland, both during and after the procedure. This is further supported by a case report by Togha et al., which described a patient developing a headache with autonomic symptoms 3 months after radiosurgery for a pituitary adenoma [8]. Furthermore, hormone secretion imbalances related to the pituitary gland can also trigger headaches [24], such as changes in prolactin secretion [25]. Based on these observations, we hypothesize that changes in prolactin secretion by the pituitary gland may be linked to headache occurrence.

Several studies have explored the link between the basal ganglia and headaches, indicating that it receives inputs from the thalamus and all cortical regions via the basal ganglia‐thalamus‐cortex circuit, playing a crucial role in the integration of sensory, emotional, and cognitive pain information [27]. Further, investigations into brain connectivity in patients with migraines have revealed significant activation in the basal ganglia region [28]. Our study found that patients with basal ganglia lesions treated with radiosurgery developed headache symptoms, suggesting that structural abnormalities in the basal ganglia, involved in pain processing, may contribute to headaches.

Our study analyzed factors linked to headaches post‐radiosurgery, the first to specifically focus on this adverse event. Female sex, younger age, no prior brain surgery, and multiple lesions significantly contributed to post‐CKRS headaches. Females exhibited a 2.2‐fold increased risk of post‐CKRS headaches (HR = 2.16), potentially linked to their higher baseline prevalence of migraine and estrogen‐mediated pain sensitization [29, 30].

Elderly patients increasingly undergo radiosurgery due to its safety and efficacy [31], with our study revealing a decreasing HARB risk with age, dropping by around 3% annually, possibly due to increased tolerance associated with aging [32].

In our study, increasing the irradiated volume did not raise headache risk, but irradiating multiple lesions did, with a 2.3 times higher risk. Current research indicates that various brain regions, such as the cortex, thalamus, hypothalamus, and brainstem, are strongly associated with the occurrence of headache [33]. Consequently, irradiating multiple lesions poses a greater likelihood of stimulating these regions and subsequently triggering headaches.

We found that prior craniocerebral surgery reduced headache risk, likely due to the reduced edema effect post‐radiosurgery [23]. The role of BED and PTV in headache occurrence post‐radiosurgery remains inconclusive, with studies showing mixed findings. For example, a study by Conti et al. found that tumor volume was an independent predictor of edema after CKRS [34], while another study did not find any correlation between PTV and hemorrhage of melanoma brain metastases after stereotactic radiosurgery [35]. In our study, neither BED nor PTV emerged as headache risk factors after stereotactic radiosurgery.

## 5 Limitations

This study has several limitations. Firstly, the exclusive focus on CKRS limits generalizability to other radiosurgery modalities (e.g., Gamma Knife). Future research should include other modalities like Gamma Knife and linear accelerator‐based radiosurgery for a fuller understanding. Secondly, longer observational studies with larger sample sizes are needed to confirm our findings and explore long‐term outcomes. Additionally, a classification system to subgroup HATRs based on headache characteristics, onset timing (immediate versus delayed), and underlying mechanisms may be warranted. Therefore, clinical trials are required to determine the most effective treatments for HARSs. Thirdly, patients undergoing radiotherapy may have different underlying causes and may have previously used various medications, such as chemotherapy drugs and molecular targeted drugs for cancer patients. Indeed, detailed follow‐up records on these medications were not obtained during our follow‐up.

## 6 Conclusion

In conclusion, in our observational nested cohort study focusing on patients who underwent CKRS, 39.9% experienced headaches post‐procedure, with an occurrence rate of 32.7%. Based on this, we proposed a new diagnostic criterion for these headaches, finding associations with sex, age, lesion count, and prior craniocerebral surgery. This highlights the significance of headaches attributed to brain radiosurgery, urging tailored treatments. While radiosurgery is safe and effective, further research is needed to understand radiation effects and develop mitigation strategies.

## Ethics Statement

The study protocol was approved by the Ethics Committee of the Chinese PLA General Hospital (ID number: S2022‐536‐01). Participants gave informed consent to participate in the study before taking part.

## Conflicts of Interest

The authors declare no conflicts of interest.

## Supporting information


Tables S1‐S4.

## Data Availability

The data generated during and/or analyzed during the current study are available from the corresponding author on reasonable request.
