# Supplementary material for: Incidence, Characteristics, and Risk Factors of Post‐Radiosurgery Headaches: A Prospective Observational Study
Source: CNS Neurosci Ther. 2025 Mar 18;31(3):e70344. doi: 10.1111/cns.70344 (PMC11915387; doi:10.1111/cns.70344)
Supplement: Supplementary file 1 — Tables S1‐S4. [file CNS-31-e70344-s001.docx]

**Table S1. Demographic and clinicopathological characteristics of patients in post-radiosurgery headache (PRH) and no PRH groups**

| Variables | Total  (n=153) | No PRH  (n=92) | PRH  (n=61) | P |
| --- | --- | --- | --- | --- |
| Gender |  |  |  |  |
| Male | 64(41.8%) | 41(44.6%) | 23(37.7%) | 0.400 |
| Female | 89(58.2%) | 51(55.4%) | 38(62.3%) |  |
| Age (years) | 47.7±14.8 | 49.7±15.4 | 44.5±13.5 | 0.034 |
| Educational level |  |  |  |  |
| ＜High school | 42(27.5%) | 23(25.0%) | 19(31.1%) | 0.404 |
| ≥High school | 111(72.5%) | 69(75.0%) | 42(68.9%) |  |
| BMI (kg/m^2^) | 24.1±3.5 | 24.2±3.1 | 23.8±4.0 | 0.412 |
| Smoking history | 41(27.0%) | 30(32.6%) | 11(18.0%) | 0.046 |
| Drinking history | 34(22.2%) | 21(22.8) | 13(21.3) | 0.825 |
| History of hypertension | 31(20.3%) | 21(22.8%) | 10(16.4%) | 0.332 |
| History of diabetes | 12(7.8%) | 10(10.9%) | 2(3.3%) | 0.161 |
| History of brain surgery | 74(45.4%) | 39(42.4%) | 35(57.4%) | 0.069 |
| Lesion number |  |  |  |  |
| Single | 116(75.8%) | 69(75.0%) | 47(77.0%) | 0.772 |
| Multiple | 37(24.2%) | 23(25.0%) | 14(23.0%) |  |
| Lesion involvement |  |  |  |  |
| Meninges | 52(34.0%) | 34(37.0%) | 18(29.5%) | 0.341 |
| Brain parenchyma | 101(66%) | 58(63.0%) | 43(70.5%) |  |

**Table S2. Characteristics of 3 different types of post-radiosurgery headache occurred at different times(N=61)**

|  | Intraoperative headache  N=2 | Interoperative headache  N=28 | Postoperative headache  N=47 | | |
| --- | --- | --- | --- | --- | --- |
|  |  |  | 1-7 days after CKRS  N=30 | 8-14 days after CKRS  N=7 | 15-90 days after CKRS  N=10 |
| Starting time^a^ |  |  |  |  |  |
| Mean | 25.0 ± 7.1 min | 4.6 ± 4.7 h | 2.6±0.4 days | 10.9±0.6 days | 45.9±4.8 days |
| Median | 25 min | 3 h | 2.0 days | 10.0 days | 50.0 days |
| IQR | N/A | 3.8 h | 2.0 days | 2.0 days | 30.0 days |
| Duration |  |  |  |  |  |
| Mean | 20.0±14.1 min | 8.4±9.6 h | 12.4±3.2 h | 8.0±4.2 h | 27.4±16.3 h |
| Median | 20 min | 4 h | 5.5 h | 3.0 h | 6.0 h |
| IQR | N/A | 20.0 h | 23.8 h | 23.8 h | 29.2 h |
| Resolution time |  |  |  |  |  |
| Mean | 45.0±21.2 min | 11.5±16.9 days | 29.9±12.0 days | 32.0±7.2 days | 52.6±16.2 days |
| Median | 45 min | 5.0 days | 12.0 days | 21.0 days | 54.0 days |
| IQR | N/A | 11.0 days | 21.8 days | 34.0 days | 26.0 days |
| Position |  |  |  |  |  |
| Unilateral | 0 | 22 (78.6%) | 24 (80.0%) | 5 (71.4%) | 6 (60.0%) |
| Bilateral | 2 (100.0%) | 6 (21.4%) | 6 (20.0%) | 2 (28.6%) | 4 (40.0%) |
| Location |  |  |  |  |  |
| Frontal | 0 | 4 (14.3%) | 5 (16.7%) | 0 | 2 (20.0%) |
| Temporal | 0 | 8 (28.6%) | 9 (30.0%) | 1 (14.3%) | 1 (10.0%) |
| Occipital | 1 (50.0%) | 8 (28.6%) | 9 (30.0%) | 1 (14.3%) | 4 (40.0%) |
| Parietal | 1 (50.0%) | 9 (32.1%) | 5 (16.7%) | 4 (57.1%) | 2 (20.0%) |
| Full head | 0 | 2 (7.1%) | 2 (6.7%) | 1 (14.3%) | 1 (10.0%) |
| Intensity (VAS) |  |  |  |  |  |
| Mean | 7.0±1.4 | 4.6±2.3 | 4.5±0.3 | 5.4±0.6 | 4.4±0.5 |
| Median | 7.0 | 4.0 | 4.0 | 5.0 | 4.0 |
| IQR | N/A | 3.0 | 3.0 | 2.0 | 3.0 |
| Quality |  |  |  |  |  |
| Throbbing | 0 | 6 (21.4%) | 8 (26.7%) | 3 (42.9%) | 1 (10.0%) |
| Dull | 0 | 18 (64.3%) | 14 (46.7%) | 3 (42.9%) | 7 (70.0%) |
| Pressing | 1 (50.0%) | 2 (7.1%) | 5 (16.7%) | 1 (14.3%) | 2 (20.0%) |
| Stabbing | 0 | 2 (7.1%) | 3 (10.0%) | 0 | 0 |
| Burning | 1 (50.0%) | 0 | 0 | 0 | 0 |
| Associated symptoms |  |  |  |  |  |
| Nausea | 1 (50.0%) | 10 (35.7%) | 8 (26.7%) | 1 (14.3%) | 1 (10.0%) |
| Vomiting | 0 | 8 (28.6%) | 4 (13.3%) | 0 | 1 (10.0%) |
| Photophobia | 0 | 3 (10.7%) | 2 (6.7%) | 1 (14.3%) | 0 |
| Phonophobia | 0 | 6 (21.4%) | 6 (20.0%) | 1 (14.3%) | 3 (30.0%) |
| Aura | 0 | 0 | 0 | 0 | 0 |

^a^ In intraoperative and interoperative headaches, the starting time was calculated from the time after the beginning of the procedure; in postoperative headaches, starting time was calculated from the time after the last fraction, as well as the resolution time; PRH, post radiosurgery headache; CKRS, CyberKnife Radiosurgery; IQR, interquartile range; NRS, numeric pain rating scale; N/A, not applicable.

**Table S3. Detailed headache characteristics for each patient**

| Patient | Starting  time^a^ | Duration | Position  Unilateral/  bilateral | Location  F/T/O/P/G^b^ | Quality  T/D/P/S/B^c^ | Intensity  (VAS) | Associated  symptoms  Nausea/Vomiting  photophobia/  phonophobia/aura | History  of HA | HA different  from  previous HA |
| --- | --- | --- | --- | --- | --- | --- | --- | --- | --- |
| 1 | 20min | 10min | －/＋ | －/－/+/－/－ | －/－/+/－/－ | 6 | －/－/－/－/－ | No |  |
| 2 | 30min | 30min | －/＋ | －/－/－/+/－ | －/－/－/$－$/+ | 8 | ＋/－/－/－/－ | No |  |
| 3 | 1h | 4h | ＋/－ | －/－/－/+/－ | +/－/－/－/－ | 3 | －/－/－/－/－ | No |  |
| 4 | 2h | 6h | ＋/－ | －/+/－/－/－ | －/+/－/－/－ | 6 | －/－/－/－/－ | Yes | Yes |
| 5 | 2h | 30min | －/＋ | －/－/+/－/－ | －/+/－/－/－ | 5 | －/－/－/－/－ | No |  |
| 6 | 2h | 8h | －/＋ | －/－/+/－/－ | －/+/－/－/－ | 3 | －/－/－/－/－ | No |  |
| 7 | 2h | 4h | ＋/－ | －/+/－/－/－ | －/+/－/－/－ | 8 | －/－/－/－/－ | No |  |
| 8 | 2h | 2min | ＋/－ | －/－/+/－/－ | +/－/－/－/－ | 4 | －/－/－/－/－ | No |  |
| 9 | 2h | 1h | ＋/－ | －/－/－/+/－ | －/+/－/－/－ | 4 | －/－/－/－/－ | No |  |
| 10 | 4h | 3h | －/＋ | －/－/－/－/+ | －/+/－/－/－ | 3 | +/+/－/+/－ | No |  |
| 11 | 6h | 1h | ＋/－ | －/－/－/+/－ | －/+/－/－/－ | 3 | －/－/－/－/－ | No |  |
| 12 | 12h | 1min | －/＋ | －/－/－/+/－ | －/－/－/+/－ | 2 | －/－/－/－/－ | No |  |
| 13 | 12h | 1h | ＋/－ | －/－/+/－/－ | －/+/－/－/－ | 3 | －/－/－/－/－ | No |  |
| 14 | 20h | 24h | ＋/－ | －/－/－/+/－ | －/+/－/－/－ | 8 | －/－/+/－/－ | No |  |
| 15 | 1 day | 3min | ＋/－ | －/－/+/－/－ | －/－/－/+/－ | 7 | －/－/－/－/－ | No |  |
| 16 | 1 day | 10min | ＋/－ | －/－/+/－/－ | －/+/－/－/－ | 3 | －/－/－/+/－ | No |  |
| 17 | 2 days | 48h | －/＋ | －/－/－/+/－ | －/－/+/－/－ | 6 | ＋/－/－/－/－ | Yes | Yes |
| 18 | 2 days | 24h | ＋/－ | －/－/－/+/－ | －/+/－/－/－ | 4 | －/－/－/－/－ | No |  |
| 19 | 2 days | 2h | ＋/－ | －/－/+/－/－ | +/－/－/－/－ | 6 | －/－/－/－/－ | No |  |
| 20 | 3 days | 4h | ＋/－ | －/－/+/－/－ | +/－/－/－/－ | 7 | －/－/－/－/－ | Yes | Yes |
| 21 | 3 days | 2min | ＋/－ | －/－/+/－/－ | －/+/－/－/－ | 5 | －/－/－/－/－ | Yes | No |
| 22 | 3 days | 8h | ＋/－ | +/－/－/－/－ | －/+/－/－/－ | 6 | －/－/－/－/－ | Yes | Yes |
| 23 | 3 days | 1min | ＋/－ | －/+/－/－/－ | +/－/－/－/－ | 3 | －/－/－/－/－ | No |  |
| 24 | 3 days | 10min | ＋/－ | －/－/+/－/－ | －/－/－/+/－ | 3 | －/－/－/－/－ | No |  |
| 25 | 3 days | 72h | ＋/－ | －/+/－/－/－ | －/－/－/+/－ | 2 | +/+/+/+/－ | No |  |
| 26 | 6 days | 2h | －/＋ | －/－/－/－/+ | +/－/－/－/－ | 4 | ＋/－/－/－/－ | No |  |
| 27 | 7 days | 3min | ＋/－ | +/－/－/－/－ | －/－/+/－/－ | 8 | －/－/－/－/－ | Yes | Yes |
| 28 | 7 days | 1min | ＋/－ | +/－/－/－/－ | +/－/－/－/－ | 4 | －/－/－/－/－ | No |  |
| 29 | 10 days | 24h | ＋/－ | －/－/－/+/－ | +/－/－/－/－ | 3 | －/－/－/－/－ | Yes | No |
| 30 | 10 days | 24h | －/＋ | －/－/－/－/+ | +/－/－/－/－ | 7 | －/－/－/－/－ | Yes | Yes |
| 31 | 10 days | 3h | －/＋ | －/－/－/+/－ | －/+/－/－/－ | 8 | －/－/+/－/－ | No |  |
| 32 | 10 days | 1h | ＋/－ | －/－/－/+/－ | －/+/－/－/－ | 5 | ＋/－/－/－/－ | No |  |
| 33 | 10 days | 4h | ＋/－ | －/－/+/－/－ | －/+/－/－/－ | 5 | －/－/－/－/－ | No |  |
| 34 | 12 days | 1min | ＋/－ | －/－/－/+/－ | +/－/－/－/－ | 5 | －/－/－/－/－ | No |  |
| 35 | 14 days | 10min | ＋/－ | －/+/－/－/－ | －/－/+/－/－ | 5 | －/－/－/+/－ | Yes | Yes |
| 36 | 26 days | 6h | ＋/－ | －/－/+/－/－ | －/－/+/－/－ | 4 | +/+/－/+/－ | No |  |
| 37 | 30 days | 1min | ＋/－ | －/－/－/+/－ | －/+/－/－/－ | 3 | －/－/－/－/－ | Yes | No |
| 38 | 30 days | 24h | －/＋ | +/－/－/－/－ | －/+/－/－/－ | 6 | －/－/－/+/－ | No |  |
| 39 | 33 days | 168h | －/＋ | －/+/－/－/－ | －/+/－/－/－ | 8 | －/－/－/－/－ | No |  |
| 40 | 40 days | 4h | －/＋ | －/－/+/－/－ | －/+/－/－/－ | 6 | －/－/－/－/－ | No |  |
| 41 | 60 days | 6h | －/＋ | －/－/－/－/+ | －/+/－/－/－ | 3 | －/－/－/+/－ | Yes | No |
| 42 | 60 days | 1min | ＋/－ | －/－/+/－/－ | －/－/+/－/－ | 3 | －/－/－/－/－ | No |  |
| 43 | 60 days | 48h | ＋/－ | －/－/－/+/－ | +/－/－/－/－ | 4 | －/－/－/－/－ | No |  |
| 44 | 60 days | 1h | ＋/－ | －/－/+/－/－ | －/+/－/－/－ | 3 | －/－/－/－/－ | No |  |
| 45 | 60 days | 17h | ＋/－ | +/－/－/－/－ | －/+/－/－/－ | 4 | －/－/－/－/－ | No |  |
| 46 | 1h | 12h | －/＋ | +/－/－/－/－ | －/+/－/－/－ | 8 | +/+/+/+/－ | No |  |
|  | 1 day | 12h | －/＋ | +/－/－/－/－ | －/+/－/－/－ | 3 | +/+/－/－/－ | No |  |
| 47 | 1h | 6h | －/＋ | －/+/－/－/－ | －/+/－/－/－ | 3 | －/－/－/－/－ | No |  |
|  | 1 day | 6h | －/＋ | －/+/－/－/－ | －/+/－/－/－ | 6 | －/－/－/－/－ | No |  |
| 48 | 1h | 4h | ＋/－ | －/+/－/－/－ | +/－/－/－/－ | 9 | +/+/－/－/－ | No |  |
|  | 5 days | 30min | ＋/－ | －/+/－/－/－ | +/－/－/－/－ | 6 | －/－/－/－/－ | No |  |
| 49 | 1h | 24h | ＋/－ | －/+/－/－/－ | －/－/+/－/－ | 3 | －/－/－/－/－ | No |  |
|  | 1 day | 24h | ＋/－ | －/+/－/－/－ | －/－/+/－/－ | 3 | －/－/－/－/－ | No |  |
| 50 | 2h | 6h | ＋/－ | －/－/－/+/－ | －/+/－/－/－ | 4 | －/－/－/－/－ | Yes | Yes |
|  | 6 days | 24h | ＋/－ | －/－/－/+/－ | －/－/+/－/－ | 4 | －/－/－/－/－ | No |  |
| 51 | 2h | 1h | ＋/－ | －/+/－/－/－ | －/+/－/－/－ | 4 | ＋/－/－/－/－ | No |  |
|  | 1 day | 1h | ＋/－ | －/+/－/－/－ | －/+/－/－/－ | 6 | －/－/－/－/－ | No |  |
| 52 | 3h | 1h | ＋/－ | －/－/+/－/－ | +/－/－/－/－ | 3 | －/－/－/－/－ | Yes | Yes |
|  | 1 day | 6h | ＋/－ | －/－/+/－/－ | －/+/－/－/－ | 3 | －/－/－/－/－ | No |  |
| 53 | 3h | 24h | ＋/－ | －/－/+/－/－ | －/+/－/－/－ | 5 | －/－/－/－/－ | Yes | No |
|  | 1 day | 5h | ＋/－ | －/－/+/－/－ | －/+/－/－/－ | 5 | －/－/－/－/－ | No |  |
| 54 | 3h | 2h | ＋/－ | +/－/－/－/－ | +/－/－/－/－ | 4 | －/－/－/－/－ | Yes | Yes |
|  | 7 days | 30min | ＋/－ | －/+/－/－/－ | +/－/－/－/－ | 2 | －/－/－/－/－ | No |  |
| 55 | 3h | 24h | ＋/－ | －/－/－/+/－ | －/+/－/－/－ | 10 | +/+/－/－/－ | No |  |
|  | 2 days | 6h | ＋/－ | －/－/－/+/－ | －/+/－/－/－ | 5 | ＋/－/－/－/－ | No |  |
| 56 | 4h | 24h | ＋/－ | +/－/－/－/－ | －/+/－/－/－ | 3 | +/+/－/－/－ | No |  |
|  | 1 day | 24h | ＋/－ | +/－/－/－/－ | －/+/－/－/－ | 3 | ＋/－/－/－/－ | No |  |
| 57 | 4h | 3min | ＋/－ | －/－/－/+/－ | －/+/－/－/－ | 2 | －/－/－/－/－ | No |  |
|  | 1 day | 3min | ＋/－ | －/－/－/+/－ | －/+/－/－/－ | 2 | －/－/－/－/－ | No |  |
| 58 | 5h | 30min | ＋/－ | +/－/－/－/－ | －/+/－/－/－ | 4 | +/－/－/+/－ | No |  |
|  | 1 day | 6h | －/＋ | －/+/－/－/－ | －/+/－/－/－ | 4 | －/－/－/+/－ | No |  |
| 59 | 6h | 6h | ＋/－ | －/+/－/－/－ | +/－/－/－/－ | 3 | +/+/－/+/－ | Yes | Yes |
|  | 1 day | 48h | ＋/－ | －/+/－/－/－ | +/－/－/－/－ | 3 | －/－/－/+/－ | No |  |
| 60 | 12h | 24h | ＋/－ | －/－/+/－/－ | －/－/－/+/－ | 5 | +/+/+/+/－ | No |  |
|  | 1 day | 24h | ＋/－ | －/－/+/－/－ | －/－/－/+/－ | 5 | +/+/+/+/－ | No |  |
| 61 | 12h | 24h | －/＋ | －/－/－/－/+ | －/－/+/－/－ | 8 | +/+/－/+/－ | Yes | No |
|  | 1 day | 24h | －/＋ | －/－/－/－/+ | －/－/+/－/－ | 8 | +/+/－/+/－ | No |  |

^a^ In intraoperative and interoperative headaches, the starting time was calculated from the time after the beginning of the procedure; in postoperative headaches, starting time was calculated from the time after the last fraction; ^b^ F/T/O/P/G=Frontal/Temporal/Occipital/Parietal/Generalized; ^c^ T/D/P/S/B=Throbbing/Dull/Pressing/Stabbing/Burning; HA: headache; VAS: visual

analogue scale.

**Table S4. Demographic and clinicopathological characteristics of patients with headache attributed to radiosurgery of the brain (HARB) and no HARB groups according to recommended diagnostic criteria in Table 2**

| Variables | Total  (n=153) | No HARB  (n=103) | HARB  (n=50) | P |
| --- | --- | --- | --- | --- |
| Gender |  |  |  |  |
| Male | 64(41.8%) | 45(43.7%) | 19(38.0%) | 0.417 |
| Female | 89(58.2%) | 58(56.3%) | 31(62.0%) |  |
| Age (years) | 47.7±14.8 | 49.4±15.0 | 44.1±14.1 | 0.041 |
| Educational level |  |  |  |  |
| ＜High school | 42(27.5%) | 27(26.2%) | 15(30.0%) | 0.623 |
| ≥High school | 111(72.5%) | 76(73.8%) | 35(70.0%) |  |
| BMI (kg/m^2^) | 24.1±3.5 | 24.2±3.3 | 23.8±4.0 | 0.514 |
| Smoking history | 41(26.8%) | 30(29.1%) | 11(22.0%) | 0.351 |
| Drinking history | 34(22.2%) | 23(22.3%) | 11(22.0%) | 0.963 |
| History of headache | 37(24.2%) | 22(21.4%) | 15(30.0%) | 0.242 |
| History of hypertension | 31(20.3%) | 22(21.4%) | 9(18.0%) | 0.628 |
| History of diabetes | 12(7.8%) | 10(9.7%) | 2(4.0%) | 0.362 |
| History of brain surgery | 74(48.4%) | 49(47.6%) | 25(50.0%) | 0.778 |
| Lesion number |  |  |  |  |
| Single | 116(75.8%) | 78(75.7%) | 38(76.0%) | 0.971 |
| Multiple | 37(24.2%) | 25(24.3%) | 12(24.0%) |  |
| Lesion involvement |  |  |  |  |
| Meninges | 52(34.0%) | 36(35.0%) | 16(32.0%) | 0.718 |
| Brain parenchyma | 101(66.0%) | 67(65.0%) | 34(68.0%) |  |
| BED (Gy) | 29.3(27.3, 37.5) | 29.3(27.3, 37.5) | 29.3(27.3, 34.8) | 0.048 |
| Brainstem average dose (Gy) | 1.7(0.9, 3.0) | 1.6(0.9, 3.2) | 1.9(1.1, 3.0) | 0.388 |
| Brainstem maximum dose (Gy) | 7.2(4.3, 18.7) | 6.6(4.3, 18.8) | 10.8(3.7, 18.8) | 0.583 |
| RBV (cm^3^) | 30.3(26.5, 33.8) | 30.8(26.8, 34.2) | 28.9±5.4 | 0.085 |
| Target average dose (Gy) | 27.5(25.1, 29.8) | 27.7(25.4, 30.4) | 27.0(24.6, 28.5) | 0.128 |
| Target maximum dose (Gy) | 32.1(30.0, 33.3) | 32.1(30.0, 34.3) | 31.6(30.0, 32.1) | 0.156 |
| PTV (cm^3^) | 5.2(1.8, 9.5) | 4.4(1.6, 8.3) | 5.4(2.3, 11.6) | 0.251 |

BED, biologically effective dose; RBV, radiation brainstem volume; PTV, planning target volume.
